# Caracterización bioquímica del veneno de la serpiente *Bothrops roedingeri* Mertens, 1942, y sus actividades edematógena, hemorrágica y miotóxica

**DOI:** 10.7705/biomedica.5228

**Published:** 2020-12-11

**Authors:** Oswaldo Nina-Cueva, Derly Olazábal-Chambilla, Jair Quispe-Arpasi, Adell Alzamora-Sánchez, Mauricio Gomes-Heleno, Salomón Huancahuire-Vega

**Affiliations:** 1 Laboratorio de Investigación en Biología Molecular, Escuela de Medicina, Facultad de Ciencias de la Salud, Universidad Peruana Unión, Lima, Perú Universidad Peruana Unión Facultad de Ciencias de la Salud Universidad Peruana Unión Lima Peru; 2 Laboratorio de Química de Proteínas, Departamento de Bioquímica, Instituto de Biología, Universidad Estatal de Campinas, Sao Paulo, Brasil Universidade Estadual de Campinas Departamento de Bioquímica Instituto de Biología Universidad Estatal de Campinas Sao Paulo Brazil

**Keywords:** Bothrops, venenos de serpiente, mordeduras de serpientes, enfermedades desatendidas, Bothrops, snake venoms, snake bites, neglected diseases

## Abstract

**Introducción.:**

El envenenamiento por mordedura de serpiente es considerado por la Organización Mundial de la Salud (OMS) una enfermedad tropical desatendida. Si bien los venenos de otras serpientes *Bothrops* se vienen estudiado ampliamente, poco se conoce del de *Bothrops roedingeri.*

**Objetivos.:**

Caracterizar bioquímicamente el veneno total de la serpiente *B. roedingeri* y evaluar su actividad miotóxica, edematógena y hemorrágica.

**Materiales y métodos.:**

Se hizo la caracterización enzimática del veneno de *B. roedingeri* determinando la actividad de la fosfolipasa A_2_ (PLA_2_) y de las enzimas proteolíticas, así como su acción fibrinogenolítica mediante electroforesis en gel de poliacrilamida con duodecilsulfato sódico *(sodium dodecyl sulfate polyacrylamide gel* electrophoresis, SDS-PAGE), y la caracterización tóxica del veneno estableciendo la dosis hemorrágica mínima, la dosis edematógena mínima y el efecto miotóxico local y sistémico.

**Resultados.:**

La actividad de las PLA_2_ del veneno total de *B. roedingeri* fue de 3,45 ± 0,11 nmoles/minuto, la proteolítica, de 0,145 ± 0,009 nmoles/minuto, en tanto que el índice de coagulación del fibrinógeno fue de 6,67 ± 1,33 segundos. Por otro lado, el veneno produjo una dosis hemorrágica mínima de 24,5 µg, una dosis edematógena mínima de 15,6 µg y un pronunciado efecto miotóxico local evidenciado por la elevación de los niveles plasmáticos de creatina cinasa después de la inoculación por vía intramuscular. No se registró miotoxicidad sistémica.

**Conclusiones.:**

El veneno de *B. roedingeri* tiene efectos hemorrágicos, edematógenos y miotóxicos locales, así como una elevada actividad de la PLA_2_, que sería responsable de los efectos miotóxico y edematógeno. También presentó actividad proteolítica, la cual podría afectar la coagulación, dada su capacidad para degradar el fibrinógeno y producir hemorragia por acción de las metaloproteasas.

## Abstract (en)

**Introduction:**

Snakebite envenoming is considered by the World Health Organization (WHO) as a neglected tropical disease. Currently, *Bothrops* snake venoms are being studied intensively, but there is little knowledge about *Bothrops roedingeri* venom.

**Objectives:**

To biochemically characterize *B. roedingeri* total venom and evaluate its myotoxic, edematogenic, and hemorrhagic activity.

**Materials and methods:**

We characterized *B. roedingeri* venom enzymatic activity by determining the phospholipase A_2_ and the proteolytic and fibrinogenolytic action using SDS-PAGE electrophoresis while we characterized its venom toxicity by determining the minimum hemorrhagic dose, the minimum edema dose, and the local and systemic myotoxic effects.

**Results:**

*Bothrops roedingeri* venom showed a PLA_2_ activity of 3.45 ± 0.11 nmoles/min, proteolytic activity of 0.145 ± 0.009 nmoles/min, and a fibrinogen coagulation index of 6.67 ± 1.33 seconds. On the other hand, it produced an minimum hemorrhagic dose of 24.5 µg, an minimum edema dose of 15.6 µg, and a pronounced local myotoxic effect evidenced by the elevation of plasma creatine kinase levels after intramuscular inoculation. The venom showed no systemic myotoxicity.

**Conclusions:**

*Bothrops roedingeri* venom has local hemorrhagic, edematogenic, and myotoxic activity. Enzymatically, it has high PLA_2_ activity, which would be responsible for the myotoxic and edematogenic effects. It also has proteolytic activity, which could affect coagulation given its ability to degrade fibrinogen, and it causes bleeding through the metalloproteases.

En junio del 2017, la Organización Mundial de la Salud (OMS) clasificó el envenenamiento por mordedura de serpiente en la categoría A de las enfermedades tropicales desatendidas. Este tipo de envenenamiento afecta a 2,7 millones de personas cada año, la mayoría de ellas viven en algunos de los lugares más remotos y menos desarrollados y pertenecen a comunidades tropicales políticamente marginadas ^1^. En Latinoamérica y el Caribe, las estadísticas hospitalarias indican que al año ocurren, por lo menos, 70.000 casos de mordeduras de serpiente, aunque es probable que el número real de envenenamientos sea mayor debido a que no se registran en muchas regiones rurales ^2^. En Perú se reportan anualmente más de 2.000 casos de ofidismo y las serpientes del género *Bothrops* son las de mayor interés médico, ya que son responsables de la mayoría de los casos de mordeduras de serpientes (70-80 %) que ocurren en el país ^3^.

*Bothrops roedingeri,* llamada "jergón de la costa" pertenece a la familia Viperidae, subfamilia Crotalinae ^4^; es la especie de jergón más pequeña, está distribuida por toda la costa sur y la sierra del Perú y a lo largo de las montañas hasta las lomas costeras, con registros desde el departamento de La Libertad hasta el sur del departamento de Arequipa ^5^. La invasión de su hábitat natural por parte de la población humana está en constante aumento, por lo que tiene un papel clave en los accidentes ofídicos en las planicies costeras del país ^5^.

Las manifestaciones generales del envenenamiento botrópico incluyen las reacciones inflamatorias graves, con daños complejos en el tejido en el que se inocula el veneno, trastornos hemostáticos drásticos, hemorragia, edema, mionecrosis y dermonecrosis, los cuales pueden complicarse con efectos sistémicos y, en algunos casos, insuficiencia renal aguda. Estas manifestaciones son el resultado de la acción de los diferentes componentes del veneno ^6^^,^^7^, cuya composición, así como las propiedades biológicas de varias de las especies de *Bothrops* en el territorio peruano *(B. atrox, B. barnetti, B. newidi, B. brazili, B. andianus,* etc.), ya se conocen ^8^^,^^9^. Sin embargo, aún no se han descrito las características ni la composición del veneno de *B. roedingeri.*

El desarrollo de la proteómica y la caracterización farmacológica de los venenos han permitido clasificar las principales familias de toxinas patógenas del veneno de las especies de *Bothrops.* Se ha determinado, por ejemplo, la presencia de fosfolipasas A_2_ (PLA_2_), que se encuentran entre las principales proteínas tóxicas del veneno botrópico y causan las reacciones clínicas más notorias en las personas envenenadas. Además, se ha evidenciado la presencia de metaloproteasas dependientes de zinc, que son los componentes más abundantes de estos venenos y causan hemorragia, de las proteasas de serina con actividad semejante a la trombina, las cuales producen alteraciones en la coagulación, y de las L-aminoácido oxidasas ^10^^,^^11^.

Las características enzimáticas del veneno, así como las propiedades farmacológicas de las serpientes endémicas del Perú, son de importancia médica, y este es el caso de *B. roedingeri,* de la cual se tiene muy poca información ^12^. En este estudio se hizo la caracterización bioquímica y enzimática del veneno de *B. roedingeri,* así como la caracterización cuantitativa de su efecto hemorrágico, edematógeno y miotóxico.

## Materiales y métodos 

### Veneno

El veneno fue cedido por el Laboratorio de Química de Proteínas del Instituto de Biología de la Universidad Estatal de Campinas (UNICAMP, São Paulo, Brasil), a cargo del profesor Sergio Marangoni. Fue obtenido de un conjunto de venenos de serpientes adultas *B. roedingeri* de ambos sexos y mantenido a 4 °C hasta el momento de usarlo diluyéndolo en 0,1 M de solución tampón fosfato (PBS) con pH de 7,2.

### Animales

Se utilizaron ratones albinos *Mus musculus* juveniles machos de la cepa BALB/c (18 a 20 g), la cual se emplea en estudios toxicológicos y farmacológicos. Los ratones provenían del Bioterio de la Escuela de Medicina Humana de la Universidad Peruana Unión (Lima, Perú) y habían sido mantenidos en ambientes con temperatura controlada, y alimentados con agua y comida *ad libitum.* El estudio fue aprobado por el Comité de Investigación y Ética de la Universidad Peruana Unión.

### *Actividad de la fosfolipasa A*_*2*_ *(PLA*_*2*_ *)*

La actividad de la PLA_2_ se determinó siguiendo la metodología de Holzer, *et al.,* modificada para microplacas de 96 pozos ^13^. Se utilizaron 200 µl de solución tampón (Tris-HCl 10 mM, CaCl_2_ 10mM y NaCl 100 mM pH 8), 20 µl del sustrato cromogénico ácido 4-nitro-(3-octanoiloxi) benzoico y 20 µl de agua o veneno total de *B. roedingeri.*

Después de la adición del veneno, la mezcla fue incubada durante 40 minutos a 37 ^o^C y las absorbancias se leyeron a intervalos de 10 minutos. La actividad enzimática expresada como velocidad inicial (V_o_) se calculó después de 20 minutos de reacción. El ensayo se hizo por triplicado, monitoreando la formación del ácido 4-nitro-(3 hidroxi) benzoico (cromóforo) a 425 nm en un lector de microplacas VersaMax 190™ (Molecular Devices, Sunnyvale, CA, USA).

### Actividad proteolítica

La actividad proteolítica se midió utilizando el sustrato N-benzoil-L-arginina-p-nitroanilida (BapNA) modificado para microplacas de 96 pozos. La mezcla de prueba contenía 45 µl de solución tampón (Tris-HCl 10 mM, pH 8,0, CaCl_2_ 10 mM, NaCl 100 mM), 180 µl de sustrato, 15 µl de agua y 5 µl de veneno total de *B. roedingeri.*

Después de la adición de la muestra, la mezcla se incubó durante 40 minutos a 37 ^o^C y las absorbancias se leyeron a intervalos de 10 minutos a 410 nm. La actividad enzimática, expresada como velocidad inicial de la reacción (V_o_), se calculó en función de la cantidad de *p*-nitroanilina liberada (14).

## SDS-PAGE

La electroforesis en gel de poliacrilamida con duodecilsulfato sódico *(sodium dodecyl sulfate polyacrylamide gel* electrophoresis, SDS-PAGE) se ajustó a la metodología de Gallagher (15). Las placas de poliacrilamida se prepararon de forma discontinua con un gel al 5 % de concentración y gel de corrida de 12,5 %. En ambos geles se añadió 20 % de SDS. La corrida se realizó en sistema doble con miniplacas SE 250 Mighty Small II™. Los marcadores de masa molecular fosforilasa B de 97 kDa, albúmina sérica bovina de 66 kDa, ovoalbúmina de 45 kDa, anhidrasa carbónica de 30 kDa, inhibidor de tripsina de soya de 20 kDa y lisozima de 14 kDa (Sigma-Aldrich Co), así como la muestra de veneno total fueron disueltos en solución tampón Tris-HCl, 0,075 M, pH 6,8, 10 % de glicerol, 4 % de SDS y 0,001 % de bromofenol. La corrida se realizó a 30 mA. Los geles se colorearon con solución azul de Coomassie al 0,05 % y el exceso de colorante se removió en ácido acético al 7 %.

### Actividad fibrinogenolítica

La acción fibrinogenolítica se determinó mezclando 20 µl de veneno total con 900 µl de solución de fibrinógeno (2mg/ml) en solución tampón Tris-HCl10 mM, pH 7,4, que contenía 10 mM de CaCl_2_ y 100 mM de NaCl a 37 ^o^C. La coagulación se expresó mediante el índice de coagulación (IC) obtenido de la ecuación IC = t^-1^ *x* 100, en la cual t^1^ corresponde al inverso del tiempo en segundos. Una unidad de actividad de coagulación de fibrinógeno se definió arbitrariamente como la cantidad de enzima capaz de coagular la solución de fibrinógeno en un minuto ^16^.

### Dosis hemorrágica mínima

La dosis hemorrágica mínima se define como la cantidad mínima de veneno (en µg de peso seco) que produce en ratones una lesión hemorrágica de 10 mm de diámetro dos horas después de ser inyectada por vía intradérmica. Se inyectaron por vía intradérmica en la piel dorsal afeitada de los cuatro ratones alícuotas de 0,1 ml de solución tampón PBS con 20, 50 y 100 µg de veneno. Los controles recibieron únicamente solución PBS.

Dos horas después, los animales se sacrificaron con halotano y su piel se removió cuidadosamente. A continuación, se midió el diámetro de las lesiones en la superficie interna de la piel en dos direcciones en ángulo recto con ayuda de pinzas y fondo de iluminación. La dosis hemorrágica mínima se calculó utilizando ecuaciones de regresión que relacionaron las dosis de veneno con los diámetros medios de las lesiones hemorrágicas ^17^.

### Dosis edematógena mínima

Se define como dosis edematógena mínima a la cantidad de veneno que induce 30 % de edema comparado con el control. La prueba se hizo según el método propuesto por Damico, *et al.*^18^. Se prepararon tres dosis de veneno total de *B. roedingeri* (10, 20 y 50 µg en 50 µl de solución PBS). Se inoculó cada solución en las almohadillas plantares del miembro posterior derecho de cuatro ratones albinos. En las del miembro izquierdo, se inocularon 50 µl de solución PBS.

Dos horas después, el volumen de la pata se evaluó con un pletismómetro. La actividad edematógena se expresó como el porcentaje de aumento de volumen de la pata derecha en comparación con la pata izquierda (control). Se utilizó la siguiente fórmula para calcular el porcentaje de edema:



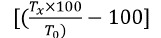



en la cual *T*_*x*_ es el edema (volumen) medido y *T*_*0*_ es el volumen de la pata antes de la inoculación del veneno.

### Actividad miotóxica local y sistémica

Los grupos de cuatro ratones recibieron una inyección intramuscular (músculo gastrocnemio) o intravenosa (vena caudal) de 20 µg de veneno total para verificar su acción local y la sistémica, respectivamente. Los grupos de control recibieron solución tampón PBS. Posteriormente, en diferentes intervalos de tiempo (1, 3, 6, 9 y 24 horas), se obtuvieron muestras de sangre de la cola en capilares heparinizados y se extrajo el plasma para determinar los niveles plasmáticos de creatina cinasa. Esta actividad se determinó mediante una prueba cinética. La actividad de la creatina cinasa se expresó en U/L y una unidad se definió como la fosforilación de 1 umol de creatina/minuto a 25 °C ^19^.

### Análisis estadístico

Las pruebas enzimáticas se realizaron por triplicado, y se calcularon la media y la desviación estándar. Para determinar la dosis hemorrágica mínima y la dosis edematógena mínima, se construyeron gráficos de dosis-respuesta con curvas de regresión lineal. Los resultados se analizaron con el programa de análisis de datos y gráficos Origin 2018™.

## Resultados

El veneno total de *B. roedingeri* se caracterizó con base en la actividad enzimática de la PLA_2_, la actividad proteolítica y la fibrinogenolítica, así como en su perfil electroforético. El valor de la actividad de PLA_2_ en una concentración de sustrato de 10 mM fue de 3,45 ±0,11 nmoles/minuto. Por otro lado, la actividad proteolítica del veneno total sobre el sustrato BApNa tuvo una V_o_ de 0,145 ± 0,009 nmoles/minuto 40 minutos después de iniciada la reacción.

**Figura 1:**
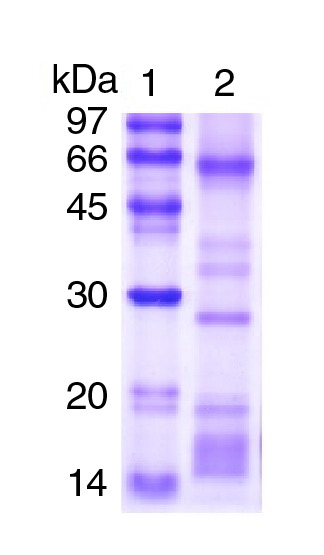
Electroforesis SDS-PAGE del veneno total de *Bothrops roedingeri.* **1)** Marcadores de masa molecular; **2)** Veneno total

La figura 1 muestra el perfil según la masa molecular de los componentes del veneno total de *B. roedingeri* (10 µg) en condiciones no reducidas. En la pista 2 se evidencia la presencia de varias bandas electroforéticas con distintas masas moleculares en relación con los marcadores de masa molecular, entre las que sobresalen las bandas de ~65, ~40, ~35, ~28 y ~19 KDa.

**Figura 2:**
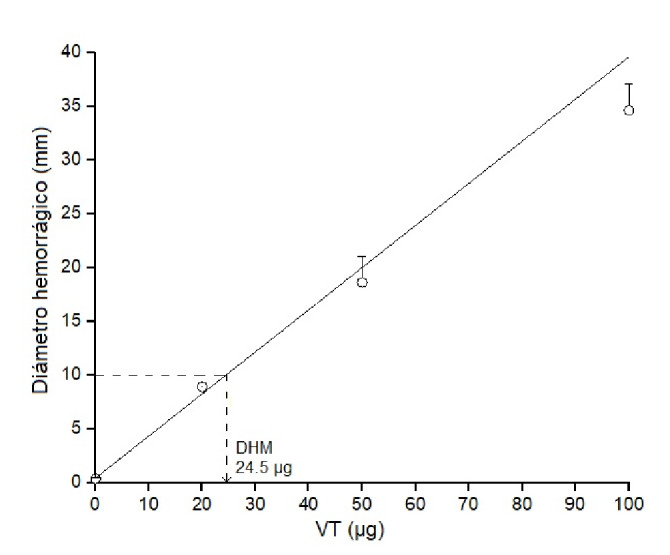
Dosis hemorrágica mínima del veneno total (VT) de *Bothrops roedingeri* en ratones. Se usaron diferentes dosis de veneno (20, 50 y 100 µg). El gráfico representa el diámetro promedio de los halos hemorrágicos ± DE (desviación estándar) (n=4). Se muestra la regresión lineal de dosis Vs. diámetros (r=0,99455).

La acción fibrinogenolítica del veneno total de *B. roedingeri* según el índice de coagulación del fibrinógeno tuvo un valor de 6,67 ± 1,33 segundos. En cuanto a la evaluación *in vivo* en ratones de la acción tóxica del veneno, la actividad hemorrágica se evidenció macroscópicamente por la presencia de halos hemorrágicos en la dermis de la región lumbar de los ratones (zona de inoculación del veneno). La dosis hemorrágica mínima fue de 24,5 µg, cantidad de veneno capaz de inducir un halo hemorrágico de 10 mm de diámetro en un tiempo de dos horas. En la figura 2 se observa que la actividad hemorrágica fue proporcional a la dosis aplicada.

**Figura 3:**
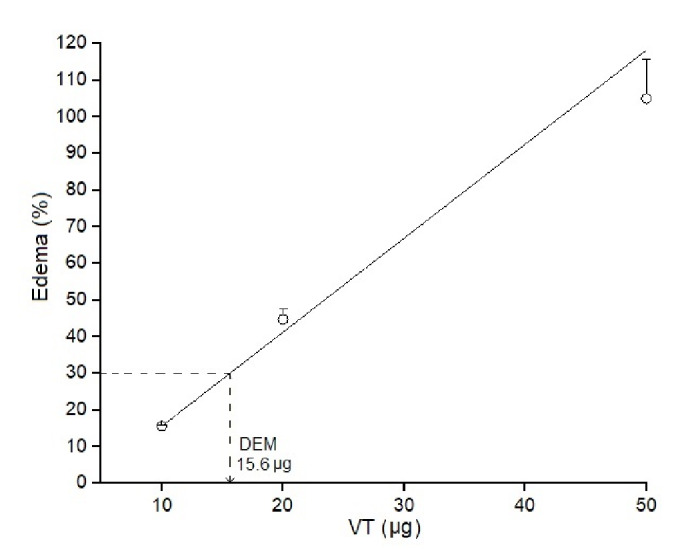
Dosis edematógena mínima del veneno total de *Bothrops roedingeri* en el modelo de pata posterior de ratones. Se usaron diferentes dosis de veneno (10, 20 y 50 µg). El grupo de control recibió solución salina (50 µl). El edema se expresa en porcentaje comparando el incremento del volumen de la pata con el respectivo control. Cada punto representa la media ± DE (desviación estándar) de cuatro ratones. Se muestra la regresión lineal de dosis Vs. porcentaje de edema (r=0,99144).

Por otro lado, la administración del veneno de *B. roedingeri* indujo la formación de edema según la dosis, lo que se evidenció macroscópicamente por el aumento del volumen del miembro posterior derecho con respecto al control (miembro posterior izquierdo). Se determinó que 15,6 µg de veneno eran capaces de inducir un 30 % de edema con respecto al control (dosis edematógena mínima) (figura 3).

**Figura 4:**
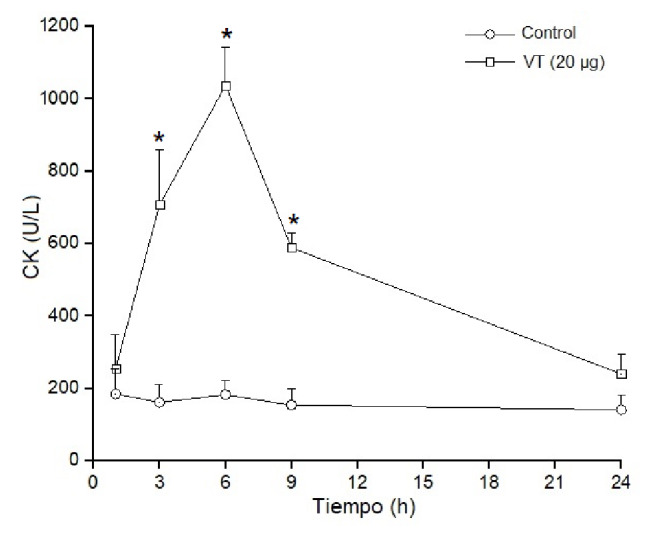
Actividad miotóxica local del veneno de *Bothrops roedingeri* en ratones medida por los incrementos en la actividad de la creatina cinasa en plasma después de la inyección intramuscular del veneno (20 µg). Los controles fueron inyectados con 50 µl de solución tampón PBS. Se obtuvo sangre periférica y se midieron los niveles séricos de creatina cinasa en diferentes momentos. Los valores se expresan como medias ± DE (desviación estándar) en cuatro ratones en cada punto (* p<0,05).

**Figura 5:**
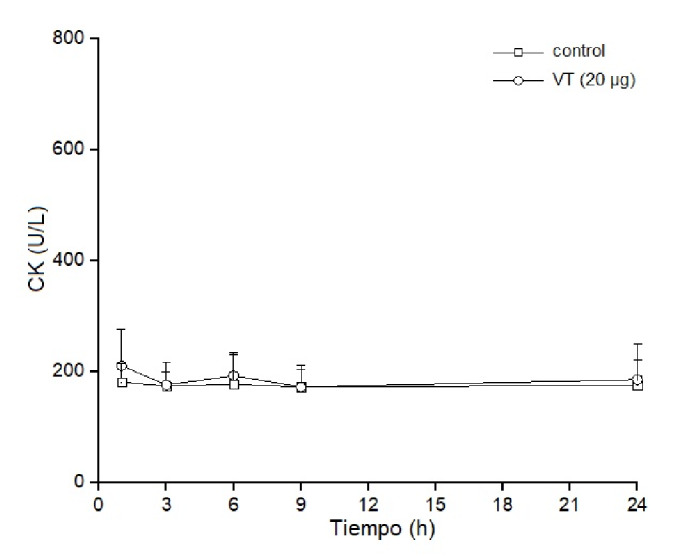
Actividad miotóxica sistémica del veneno de *Bothrops roedingeri* en ratones medida por los incrementos en la actividad de la creatina cinasa en plasma después de la inyección intravenosa del veneno (20 µg). Los controles recibieron 50 µl de solución tampón PBS. Se obtuvo sangre periférica y se midieron los niveles séricos de cretaina cinasa en diferentes momentos. Los valores se expresan como medias ± DE (desviación estándar) de cuatro ratones en cada punto (* p<0,05).

Los estudios para determinar *in vivo* el efecto miotóxico local del veneno total (20 µg) se hicieron en ratones inoculados con el veneno por vía intramuscular. El daño muscular se evaluó por el aumento de los niveles de creatina cinasa plasmática. Los resultados evidenciaron que dichos niveles aumentaron drásticamente en las primeras 3 a 6 horas de la inoculación, superando las 1.000 U/L de creatina cinasa, y volvieron a sus valores normales 24 horas después del inicio del análisis (figura 4). Por otro lado, el veneno no tuvo una actividad miotóxica significativa cuando se inoculó por vía endovenosa, y los niveles de creatina cinasa plasmática en los diferentes intervalos de tiempo fueron muy semejantes a los de los controles, lo que evidenció la ausencia de actividad miotóxica sistémica (figura 5). Las actividades tóxicas del veneno de *B. roedingeri* se compararon con las de otros venenos botrópicos de serpientes de Perú (cuadro 1).

**Cuadro 1:** Comparación entre las actividades tóxicas de *Bothrops roedingeri* y otros venenos botrópicos

Veneno	DHM	DEM	AC	AM
	(µg)	(µg)	(s)	(µg)
*Bothrops roedingeri*	24,5	15,6	6,67	20,0
*Bothrops atroxa*	4,10	2,95	3,65	56,3
*Bothrops brazilia*	3,71	1,18	7,39	13,5
*Bothrops pictusa*	1,06	0,52	46,6	15,4
*Bothrops barnettia*	0,75	1,13	21,5	40,4

DHM: dosis hemorrágica mínima, definida como la cantidad de veneno en µg que induce una lesión hemorrágica de 10 mm de diámetro dos horas después de su aplicación; DEM: dosis edematógena mínima, definida como la cantidad de veneno en µg que induce 30 % de edema con respecto al control; AC: actividad coagulante expresada como índice de coagulación en segundos; AM: actividad miotóxica expresada en términos de dosis miotóxica mínima, definida como la cantidad de veneno en µg que induce un incremento de la creatina cinasa plasmática tres horas después de la inyección intramuscular correspondientes a cuatro veces el valor de creatina cinasa de los controles.

^a^ Datos obtenidos de Rojas, *et al.*^25^

## Discusión

Las manifestaciones biológicas de los efectos tóxicos y farmacológicos que interfieren en los procesos fisiológicos normales de las presas o víctimas de mordeduras de serpiente, se relacionan con la abundancia de las diferentes familias de toxinas y de sus múltiples isoformas, las cuales actúan de forma sinérgica potenciando su toxicidad ^20^. En este estudio, se hizo la caracterización bioquímica y enzimática del veneno de la serpiente *B. roedingeri,* así como la caracterización cuantitativa de su efecto hemorrágico, edematógeno, fibrinogenolítico y miotóxico.

El veneno tuvo una apreciable actividad de PLA_2_, similar a la mostrada por otros venenos botrópicos y medida usando el mismo sustrato ^8^^,^^21^^,^^22^. Las PLA_2_ presentan masas moleculares de ~14 kDa que, en su estado nativo, tienden a encontrarse en estado dimérico. En la figura 1 se observa una banda proteica notoria de ~28 kDa, evidencia de la presencia de estas enzimas en el veneno. Por otro lado, el veneno tuvo acción proteolítica en el sustrato BApNA, lo que evidencia la actividad proteolítica de la serina proteasa. Estas proteínas del veneno tienen masas moleculares entre 30 y 35 kDa ^23^ y, según el perfil electroforético, estarían presentes en el veneno de *B. roedingeri* (figura 1). Se sabe que las serina proteasas del veneno afectan el sistema de coagulación al quebrar el fibrinógeno en sus fibrinopéptidos promoviendo la coagulación, por lo que se las conoce como "similares a trombina" ^16^^,^^23^, efecto propiciador de la coagulación que también fue observado en el veneno de *B. roedingeri,* así como en otros venenos de especies *Bothrops* que habitan en Perú (cuadro 1).

La actividad hemorrágica de los venenos botrópicos varía de acuerdo con la región, la ontogenia y, específicamente, la especie ^24^. Otro ejemplo de serpiente endémica de la selva peruana es la serpiente *B. brazili,* cuya dosis hemorrágica mínima se determinó tanto en el veneno crudo como en la proteína aislada, con valores de 3,71 y 6,61 µg, respectivamente ^25^^,^^26^. Por otro lado, utilizando la misma metodología en la serpiente *B. atrox,* se registraron valores de 4,10 ± 0,64 µg en veneno total ^25^^,^^27^. El valor de la dosis hemorrágica mínima de *B. roedingeri* fue de 24,5 µg (figura 2), valor que demuestra una actividad moderada si se la compara con las dosis hemorrágicas mínimas de estas especies botrópicas (cuadro 1).

Se sabe que, en gran parte, las responsables del efecto hemorrágico del envenenamiento ofídico son las metaloproteasas dependientes de zinc, componentes abundantes en los venenos de las serpientes botrópicas. Estas metaloproteasas se subdividen en las clases P-I, P-II y P-III según su estructura, con masas moleculares de 20-30, 30-60 y 60-100 kDa, respectivamente ^28^. Como se observa en el perfil electroforético en la figura 1, en el veneno de *B. roedingeri* las clases P-I y P-III (masas moleculares relativas de ~28 y ~66 kDa) serían las de mayor presencia. Se ha demostrado que estas clases de metaloproteasas son capaces de degradar el colágeno de tipo IV y la laminina en la lámina basal de la microvasculatura, además de quebrar la barrera endotelial e incrementar la permeabilidad vascular ^29^.

La inflamación es una característica local importante del envenenamiento por serpientes botrópicas; se caracteriza por edema local prominente, dolor e hinchazón extensa ^30^, por los que se evaluó la reacción inflamatoria local producida por el veneno total de *B. roedingeri,* verificando la formación de edema en las patas de los ratones. Los resultados evidenciaron que el veneno indujo un edema pronunciado (figura 3) de forma similar a la de otros venenos botrópicos (cuadro 1), incluidos los de *B. brazili, B. barnetti, B. atrox y B. rhombeatus*^25^^,^^31^. La exudación de fluidos y proteínas plasmáticas que conducen a la formación del edema es producida por diferentes mediadores que actúan aumentando la permeabilidad microvascular; entre ellos están, en primer lugar, la PLA_2_ y las metaloproteasas, sin embargo, las serina proteasas y las lectinas también contribuyen a las propiedades edematógenas del veneno total ^30^.

La contribución de las metaloproteasas de la clase P-I al efecto inflamatorio del veneno fue demostrada por Torres-Huaco, *et al.*^32^, cuando la toxina BmHF-1 aislada de *B. marajoensis* produjo edema en el mismo modelo animal con una dosis edematógena mínima de 10,27 µg. Por otro lado, las PLA_2_ también tienen un papel importante en los procesos inflamatorios al proporcionar los precursores de las sustancias lipídicas proinflamatorias derivados del ácido araquidónico ^33^. Además, en el veneno en estudio se aisló la PLA_2_ BrTX-I, una toxina catalíticamente activa, la cual produjo edema en las patas de ratones y aumentó los niveles séricos de IL-1, IL-6 y TNF-α, citocinas involucradas en la regulación de la reacción inmunitaria y la inflamación ^12^.

La miotoxicidad se define como la habilidad de las toxinas para inducir necrosis en la musculatura esquelética *in vivo* a partir de la inoculación intramuscular, o *in vitro* mediante la incubación con músculos esqueléticos diferenciados, y se evidencia por la capacidad de aumentar los niveles plasmáticos de creatina cinasa ^19^. Los componentes del veneno botrópico responsables de la acción miotóxica son las PLA_2_ y las metaloproteasas hemorrágicas ^34^, presentes ambas en el veneno de *B. roedingeri.* La falta de oxígeno debida a la hemorragia producida por las metaloprotesas se considera la causa de la mionecrosis ^35^; por otro lado, las PLA_2_ miotóxicas se cuentan entre los principales factores responsables de la necrosis de la musculatura esquelética en casos de envenenamiento por serpientes. Se sabe que ello ocurre debido a la unión de estas miotoxinas a la membrana plasmática de las fibras musculares, lo que produce alteraciones en su permeabilidad ^19^.

El veneno total de *B. roedingeri* aumentó drásticamente la concentración plasmática de creatina cinasa después de inyectarse en el músculo gastrocnemio de los ratones (figura 4). Este aumento de la creatina cinasa evidencia un daño muscular grave en las fibras musculares inoculadas con las toxinas, así como su efecto miotóxico local. Por el contrario, cuando el veneno se administra por vía intravenosa, la concentración plasmática de la creatina cinasa es semejante a la de los controles, lo cual demuestra la falta de miotoxicidad sistémica (figura 5).

La miotoxicidad local es característica del veneno de las serpientes Viperidae, cuyas PLA_2_ afectan predominantemente los músculos localizados en la región donde el veneno se inyecta ^8^^,^^12^^,^^33^. Estas miotoxinas se caracterizan por inducir daño muscular localizado, son de acción rápida y es poca su miotoxicidad después del daño inicial; probablemente no tienen especificidad, ya que se unen a diferentes tipos celulares, musculares y no musculares, en el lugar de la inyección y podrían ser rápidamente secuestradas por los tejidos, lo que impide que alcancen tejidos distantes del lugar de la inoculación ^19^.

En conclusión, los resultados descritos aquí demuestran que el veneno de *B. roedingeri* tiene acción hemorrágica, edematógena y miotóxica local. Enzimáticamente, el veneno tiene una acentuada actividad de PLA_2_, que sería responsable de los efectos miotóxico e inflamatorio. El veneno también es moderadamente proteolítico, lo cual podría afectar la coagulación por su capacidad para degradar el fibrinógeno, y producir hemorragia y miotoxicidad por acción de las metaloproteasas.

Los futuros estudios de neutralización con sueros antiofídicos disponibles en el país podrían contribuir a mejores tratamientos y a dilucidar el mecanismo de envenenamiento de esta serpiente de importancia médica en el Perú.
